# Pleuroparenchymal Fibroelastosis Complicated by Pulmonary Alveolar Proteinosis After Peripheral Blood Stem Cell Transplantation

**DOI:** 10.1002/rcr2.70536

**Published:** 2026-03-09

**Authors:** Keishi Sugino, Hirotaka Ono, Mikako Saito, Miho Kobayashi, Seiji Igarashib, Akira Hebisawa

**Affiliations:** ^1^ Department of Respiratory Medicine Tsuboi Hospital Koriyama Japan; ^2^ Department of Diagnostic Pathology Tsuboi Hospital Koriyama Japan; ^3^ Department of Pathology Tokyo National Hospital Tokyo Japan

**Keywords:** haematopoietic stem cell transplantation, interstitial lung disease, peripheral blood stem cell transplantation, Pleuroparenchymal fibroelastosis, pulmonary alveolar proteinosis

## Abstract

A 52‐year‐old man with a history of Philadelphia chromosome‐positive acute lymphoblastic leukaemia underwent peripheral blood stem cell transplantation (PBSCT) 10 years earlier. Five years after transplantation, he developed a persistent dry cough and exertional dyspnea. High‐resolution CT revealed upper lobe‐predominant subpleural fibrosis compatible with pleuroparenchymal fibroelastosis (PPFE) and lower lobe reticulation with ground‐glass opacities. Video‐assisted thoracoscopic lung biopsy showed subpleural fibroelastosis and bronchocentric lesions, along with intra‐alveolar accumulation of foamy macrophages containing cholesterol crystals and periodic acid–Schiff‐positive material, with surfactant protein A immunoreactivity—consistent with pulmonary alveolar proteinosis (PAP). Despite corticosteroids, tacrolimus, and subsequent antifibrotic therapy with nintedanib, the disease gradually progressed, and the patient ultimately died of acute exacerbation triggered by SARS‐CoV‐2 infection. This case highlights a rare coexistence of PPFE and PAP after PBSCT and underscores the potential role of chronic graft‐versus‐host disease and immune dysregulation in their pathogenesis.

## Graphical abstract

We describe a unique case of concomitant pleuroparenchymal fibroelastosis and pulmonary alveolar proteinosis following allogeneic peripheral blood stem cell transplantation for Philadelphia chromosome‐positive acute lymphoblastic leukaemia. This case illustrates the complex interplay of immune dysregulation, surfactant metabolism and chronic graft‐related lung injury.

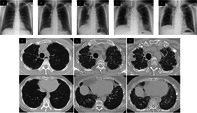

## 1 Introduction

Late‐onset non‐infectious pulmonary complications (LONIPCs) encompass various chronic lung disorders that arise more than 100 days after haematopoietic stem cell transplantation (HSCT) and are commonly associated with chronic graft‐versus‐host disease (cGVHD), such as bronchiolitis obliterans syndrome (BOS), organising pneumonia (OP), and pleuroparenchymal fibroelastosis (PPFE) [1]. Importantly, PPFE is not exclusively attributable to cGVHD, as some cases have been reported after autologous HSCT or even following chemotherapy alone [2].

Pulmonary alveolar proteinosis (PAP) is a rare disorder characterised by intra‐alveolar accumulation of surfactant‐derived material, often due to impaired alveolar macrophage clearance. Secondary PAP may arise in hematologic malignancies or post‐transplant immunosuppression [3].

Here, we describe a unique case of concomitant PPFE and PAP following allogeneic peripheral blood stem cell transplantation (PBSCT) for Philadelphia chromosome‐positive acute lymphoblastic leukaemia. This case illustrates the complex interplay of immune dysregulation, surfactant metabolism and chronic graft‐related lung injury.

## 2 Case Report

A 52‐year‐old Japanese man, an ex‐smoker, was referred to our hospital with a five‐year history of persistent dry cough and progressive exertional dyspnea. The patient had worked primarily as an office employee and had no history of occupational or environmental dust exposure. Ten years before, he had been diagnosed with Philadelphia chromosome‐positive acute lymphoblastic leukaemia (Ph + ALL) and received imatinib‐based chemotherapy following the JALSG Ph + ALL208IMA protocol. Induction and consolidation therapies were administered with continuous imatinib combined with multi‐agent chemotherapy, followed by planned imatinib maintenance. As a conditioning regimen prior to allogeneic PBSCT, the patient received total body irradiation (TBI), etoposide and cyclophosphamide. His post‐transplant course was uneventful for several years under maintenance immunosuppression with tacrolimus (TAC) and low‐dose prednisolone (PSL). Despite continuation of immunosuppressive therapy, his respiratory symptoms gradually worsened.

**FIGURE 1:**
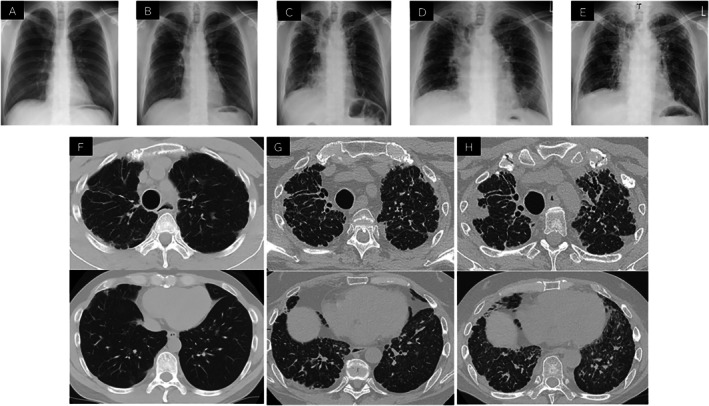
Time course of chest x‐ray and computed tomography findings. (A) No apparent abnormalities were observed on chest x‐ray at the time of PBSCT. (B) Three years after transplantation, chest x‐ray revealed mild bilateral upper lung field–predominant subpleural opacities. (C) Ten years after transplantation, at referral to our hospital, subpleural infiltrative opacities predominantly in the bilateral upper lung fields with marked volume loss and rightward tracheal shift were noted, along with reticular opacities and volume loss in the bilateral lower lung fields, predominantly on the right. (D) At 11years post‐transplantation and one year after referral to our hospital, further progression of rightward tracheal deviation was noted, along with newly developed infiltrative opacities in the left lower lung field. (E) At 11 years post‐transplantation and 2 years after referral to our hospital, chest x‐ray obtained during an acute exacerbation triggered by SARS‐CoV‐2 infection revealed newly developed bilateral ground‐glass opacities and pneumomediastinum. (F) Five years after transplantation, chest CT revealed linear lesions arising from the subpleural regions predominantly in the bilateral upper lobes, accompanied by atelectatic consolidation. (G) Ten years after transplantation, at the time of referral to our hospital, chest CT showed progression of linear lesions and atelectatic consolidation in the bilateral upper lobes, accompanied by traction bronchiectasis and marked volume loss. In addition, granular opacities and interlobular septal thickening were observed predominantly in the right lower lobe. (H) Twelve years after transplantation and 2 years after referral to our hospital, chest CT demonstrated further progression of traction bronchiectasis and atelectatic consolidation in the bilateral upper lobes, with marked volume loss. In addition, patchy ground‐glass opacities were newly observed in both lungs.

Chest x‐ray showed no apparent abnormalities at the time of PBSCT; however, mild bilateral upper lung field–predominant subpleural opacities became evident 3 years after transplantation (Figure 1A,B). Serial chest x‐ray demonstrated a gradual progression of upper‐lobe–predominant subpleural abnormalities over time (Figure 1C–E). Five years after transplantation, computed tomography demonstrated subpleural‐based linear lesions predominantly in the bilateral upper lobes with associated atelectatic consolidation (Figure 1F). At referral to our hospital, 10 years after transplantation, these abnormalities had progressed, with the development of traction bronchiectasis and marked volume loss in the bilateral upper lobes, as well as granular opacities and interlobular septal thickening predominantly in the right lower lobe (Figure 1G). Pulmonary function test (PFT) performed at the time of the patient's initial presentation revealed severe restrictive ventilatory impairment. The forced vital capacity (FVC) was 1.6 L (40.2% predicted), the forced expiratory volume in 1 s (FEV_1_) was 1.26 L (37.4% predicted), and the forced expiratory ratio (FEV_1_/FVC) was 78.6%, which was within the normal range. The diffusing capacity for carbon monoxide (DLco) was 47.1% predicted. Bronchoalveolar lavage fluid revealed an elevated percentage of lymphocyte (28%) without evidence of infection, and showed no remarkable macroscopic abnormalities.

**FIGURE 2:**
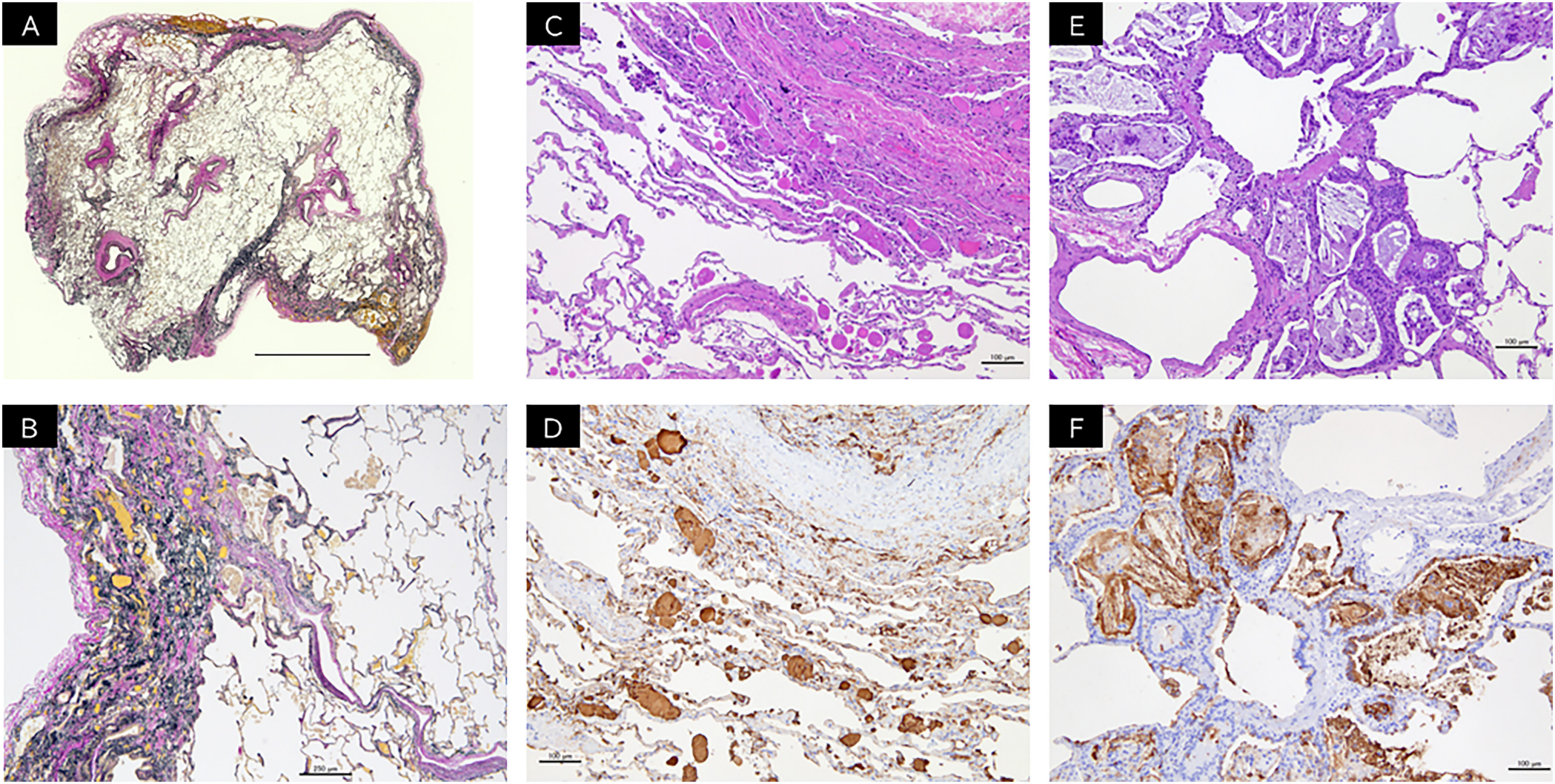
Histopathological findings of the VATS lungs. (A) Low‐power view of the left upper‐lobe specimen showing dense subpleural fibrosis with abrupt transition to relatively preserved parenchyma (Elastica van Gieson stain [EvG]) (scale bar = 1 cm). (B) Histological examination of the upper lobe specimen showed marked subpleural fibrosis with dense elastic fibre deposition, consistent with PPFE (EvG stain) (scale bar = 250 μm). (C) Eosinophilic material suggestive of surfactant was observed within and adjacent to alveolar spaces containing clusters of histiocytes (haematoxylin and eosin [HE] stain) (scale bar = 100 μm). (D) Immunohistochemistry for surfactant protein A (SP‐A) revealed diffuse cytoplasmic positivity in alveolar contents and macrophages, confirming the diagnosis of pulmonary alveolar proteinosis (SP‐A stain) (scale bar = 100 μm). (E) Foamy macrophages and histiocytes containing cholesterol crystals clustered within the respiratory bronchioles and surrounding alveolar spaces, with adjacent alveolar septa showing myxoid or fibrotic thickening and mild small round cell infiltration (HE stain) (scale bar = 100 μm). (F) These histiocytes and foamy macrophages were positive for SP‐A (SP‐A stain) (scale bar = 100 μm).

Given the progressive nature of his disease and the need for histopathological confirmation, video‐assisted thoracoscopic lung biopsy was performed. Histological examination of the upper lobe specimen showed marked subpleural fibrosis with dense elastic fibre deposition, consistent with PPFE (Figure 2A,B). In the lower lobe, bronchocentric interstitial lesion was observed, along with intra‐alveolar clusters of foamy and cholesterol crystal‐laden macrophages. These histiocytes contained periodic acid–Schiff (PAS)‐positive material, and spherical surfactant deposits within the alveolar spaces were immunohistochemically positive for surfactant protein A (SP‐A) (Figure 2C–F). No infectious organisms or granulomas were identified.

Based on these findings, a diagnosis of PPFE with secondary PAP following PBSCT was established, with negative anti–granulocyte–macrophage colony‐stimulating factor (GM‐CSF) antibodies, excluding autoimmune PAP. At the time PAP was diagnosed, there was no evidence of secondary myelodysplastic syndrome or relapse of Ph + ALL. Nintedanib was initiated for progressive fibrosing interstitial lung disease based on worsening respiratory symptoms, radiological progression and objective physiological decline. PFT performed at the referring hospital one year before presentation showed a FVC of 1.89 L (45.9% predicted), which subsequently declined to 1.6 L (40.2% predicted) at the time of initial evaluation at our hospital, supporting the presence of progressive fibrosing disease. Although corticosteroids, tacrolimus, and antifibrotic therapy with nintedanib were administered, both respiratory failure and CT findings progressively worsened. The impact of nintedanib on the subsequent rate of FVC decline could not be evaluated because serial PFT was not feasible due to the patient's progressive clinical deterioration (Figure 1H). Two years later, SARS‐CoV‐2–associated acute exacerbation resulted in fatal respiratory failure despite intensive care.

## 3 Discussion

Late‐onset non‐infectious pulmonary complications (LONIPCs) comprise a heterogeneous group of chronic lung disorders that develop more than 100 days after HSCT. These complications are widely regarded as pulmonary manifestations of cGVHD and include BOS, OP, and PPFE [1]. In our patient, histological evaluation showed no evidence of bronchiolitis obliterans. Factors other than graft‐versus‐host immune reactions, such as total body irradiation, chemotherapeutic exposure and post‐transplant immunosuppression, may also play a role in the pathogenesis of PPFE after HSCT [4].

Multiple factors may have contributed to the development of secondary PAP in this case. First, the underlying disease, ALL, may have caused quantitative and qualitative dysfunction of alveolar macrophages through bone marrow lesions and prior chemotherapy, leading to impaired surfactant clearance. Hematologic disorders are widely recognised as a major underlying condition for secondary PAP, supporting the involvement of this mechanism [5]. Secondary PAP associated with hematologic disorders is considered to result primarily from numerical and/or functional impairment of monocyte‐derived alveolar macrophages rather than from intrinsic abnormalities of surfactant production [6]. Prior cytotoxic chemotherapy and bone marrow dysfunction may interfere with macrophage differentiation and function, leading to defective surfactant catabolism. In addition, the absence of anti–GM‐CSF antibodies in the present case supports a diagnosis of secondary rather than autoimmune PAP.

Second, the presence of PPFE may have further promoted the accumulation of surfactant within the alveoli. The progressive fibrotic contraction and distortion of lung architecture associated with PPFE can impair alveolar ventilation and macrophage‐mediated surfactant processing, potentially facilitating secondary surfactant retention within the alveolar spaces. In this context, PPFE is more likely to have acted as a contributing or exacerbating factor for intra‐alveolar surfactant accumulation rather than as a primary driver of PAP.

Third, PBSCT itself may have contributed to the development of secondary PAP. After transplantation, immune dysregulation and prolonged immunosuppressive therapy can impair the recovery and function of alveolar macrophages, resulting in insufficient surfactant clearance and subsequent intra‐alveolar accumulation [3]. These post‐transplant factors may further compromise alveolar macrophage maturation and functional recovery during immune reconstitution, thereby augmenting the risk of secondary PAP.

Based on the above, the secondary PAP in this case was likely not attributable to a single factor, but rather represented the combined effects of the underlying hematologic disease, prior cytotoxic therapy, and post‐transplant immune dysfunction, including impaired immune reconstitution and prolonged immunosuppression, as the principal mechanisms, with PPFE‐associated structural lung damage acting as an additional exacerbating factor.

## Author Contributions

K.S. contributed primarily to the conception of the case report, clinical management of the patient, and drafting of the manuscript. M.S. and H.O. also contributed to the conception of the case report and clinical management. S.I., M.K. and A.H. reviewed the pathological findings and contributed to the discussion. A.H. supervised the project, critically revised the manuscript for important intellectual content and approved the final version. All authors read and approved the final version of the manuscript.

## Funding

The authors have nothing to report.

## Ethics Statement

Ethical approval for case reports is not required in our hospital's policy.

## Consent

The authors declare that written informed consent was obtained for the publication of this manuscript and accompanying images using the consent form provided by the Journal.

## Conflicts of Interest

The authors declare no conflicts of interest.

## Data Availability

The data that support the findings of this study are available on request from the corresponding author. The data are not publicly available due to privacy or ethical restrictions.
